# A reductionist, *in vitro* model of environmental circadian disruption demonstrates SCN-independent and tissue-specific dysregulation of inflammatory responses

**DOI:** 10.1371/journal.pone.0217368

**Published:** 2019-05-28

**Authors:** Adam Stowie, Ivory Ellis, Kandis Adams, Oscar Castanon-Cervantes, Alec J. Davidson

**Affiliations:** Department of Neurobiology, Neuroscience Institute, Morehouse School of Medicine, Atlanta, Georgia, United States of America; University of Texas Southwestern Medical Center, UNITED STATES

## Abstract

Environmental circadian disruption (ECD), characterized by repeated or long-term disruption in environmental timing cues which require the internal circadian clock to change its phase to resynchronize with the environment, is associated with numerous serious health issues in humans. While animal and isolated cell models exist to study the effects of destabilizing the relationship between the circadian system and the environment, neither approach provides an ideal solution. Here, we developed an *in vitro* model which incorporates both elements of a reductionist cellular model and disruption of the clock/environment relationship using temperature as an environmental cue, as occurs *in vivo*. Using this approach, we have demonstrated that some effects of *in vivo* ECD can be reproduced using only isolated peripheral oscillators. Specifically, we report exaggerated inflammatory responses to endotoxin following repeated environmental circadian disruption in explanted spleens. This effect requires a functional circadian clock but not the master brain clock, the suprachiasmatic nucleus (SCN). Further, we report that this is a result of cumulative, rather than acute, circadian disruption as has been previously observed *in vivo*. Finally, such effects appear to be tissue specific as it does not occur in lung, which is less sensitive to the temperature cycles employed to induce ECD. Taken together, the present study suggests that this model could be a valuable tool for dissecting the causes and effects of circadian disruption both in isolated components of physiological systems as well as the aggregated interactions of these systems that occur *in vivo*.

## Introduction

The circadian system is a highly conserved biological timing system in organisms from single cell bacteria to humans. In mammals, cell-autonomous clocks are present in most cells but are synchronized by a central master clock, the suprachiasmatic nucleus (**SCN**) of the anterior hypothalamus. Most physiological and behavioral processes are subject to circadian regulation which temporally optimizes the internal environment and allows anticipation of recurrent events in the external environment. Disruption of the circadian system in humans, as occurs during chronic shift work or multiple time-zone travel, has been linked to increased incidence and severity of several cancers [1–3], autoimmune disorders [4], obesity [5], diabetes [6], and stroke [7]. Animal models of shiftwork have also been employed to demonstrate the deleterious effects of circadian disruption [8, 9]. Though the mechanism(s) mediating the development of so called “shift work diseases” is not presently known, one potential explanation is that immune function is directly impacted by misalignment of circadian clocks with the external environment.

There is ample evidence of the importance of circadian regulation in the immune system. In mice, inflammation triggered by bacterial endotoxin (lipopolysaccharide (LPS)) fluctuates in a circadian manner [10], as does mortality [11]. The inflammatory response to intraperitoneally administered LPS is primarily mediated by macrophages, which have been shown to have a strong circadian expression of core clock genes [12, 13]. Specific deletion of clock genes in myeloid cells has been shown to have a significant impact on immune specific processing in these cells, either eliminating the rhythm in LPS response entirely [13] or substantially altering it [13–15]. Further, other aspects of immune cell function such as cell trafficking have been demonstrated to be regulated by circadian rhythms [16]. This evidence suggests an important role of immune dysregulation in mediating the significant health impacts of circadian disruption.

The study of the effects of circadian disruption is, however, complicated by several important factors. Inability to control for immunologically relevant behavioral and environmental factors make conducting these studies in humans very challenging, though some studies have been able to demonstrate immune consequences of environmental circadian disruption directly on the immune system, or through other physiological processes that indirectly affect inflammation [17–22]. Mammalian animal models provide better environmental control [8, 9], however the hierarchical organization of the circadian system, in which a master pacemaker resets other brain clocks which in turn reset clocks throughout the body, as well as the diffuse nature and inter-connectedness of the immune system represent significant obstacles to identifying a mechanism of circadian disruption. Cell lines provide an excellent reductionist approach to this problem, allowing for direct alteration of the genetic components of the clock and subsequent effect on cellular function in a precise, cell-type specific fashion. However, deletion of these genes breaks the clock rather than alters its relationship with the environment (as *in vivo*). Furthermore, disruption of clock genes, both *in vivo* and *in vitro*, also modifies immunological function in ways which may be distinct from the roles these genes play in rhythmicity. For example, core clock genes *clock* and both *cryptochrome 1* and *cryptochrome 2* may directly regulate expression of NF-κB [15, 23]. In such cases, clock specific effects on immune function are obscured by direct immune specific regulation by these genes. Others have previously used primary culture to demonstrate both rhythmicity *in vitro* as well as temperature sensitivity in some mammalian tissues [24–26], suggesting that a reductionist model of environmental control is entirely feasible.

Here, we a developed such an *in vitro* model of environmental circadian disruption (**ECD**) in which explanted spleen and lung were cultured in circadian high/low temperature cycles which were either kept constant or repeatedly advanced to investigate the consequences of repeated phase shifting on peripheral tissue function. This approach provides a reductionist application in which the disruption which takes place is of the environment rather than of the circadian clock, an important distinction. Further, organotypic, rather than cellular, cultures retain multi-oscillatory connectivity of diverse cell-types which is critical for normal circadian rhythms *in vivo*. In combination, these factors may represent a model of ECD which is more closely analogous to *in vivo* circadian disruption and results in immune dysregulation similar that what has been previously reported in animal models of ECD. Here, we have used this model to demonstrate that the immune-specific effects of ECD previously reported in animal models is a local phenomenon which does not require input from the master pacemaker in the SCN. Specifically, explanted spleen tissue subjected to at least four consecutive 6-hour phase advances and challenged with LPS undergo significantly increased transcription and secretion of the pro-inflammatory cytokine IL-6, as we have previously reported using *in vivo* ECD [8]. Importantly, this effect was abolished in BMAL1KO mouse spleen explants which do not possess a functional circadian clock. Exposure to fewer phase advances (less disruption) also abolished exaggerated pro-inflammatory signaling. Interestingly, advancing temperature cycles did not impact the circadian clock, nor cause a differential response to immune challenge by LPS in lung explants. Thus, the immune consequences of environmental circadian disruption can be elicited by disrupting peripheral rhythms in a reductionist model amenable to mechanistic dissection.

## Materials and methods

### Animals

Male *Per2*^*Luc*^ knock-in mice on a C57BL/6 background [27] or B6.129-Arntl^*tm1Bra*^/J (Jax 009100), 6–8 weeks old were used. Per2^Luc^ mice were obtained from the Menaker lab at UVA in 2006 and maintained as an inbred colony at Morehouse School of Medicine. Heterozygous B6.129-Arntl^*tm1Bra*^/J male and female mice were purchased from Jackson labs and bred to obtain homozygous BMAL1 KO mice, confirmed by genotyping (www.MouseGenotype.com), for the present experiments. Mice were group housed in polycarbonate cages (19.56 x 30.91 x 12.34 cm) with corncob bedding under a 12:12 LD cycle and cared for by Morehouse School of Medicine Animal Care staff. Prior to organ collection, mice were euthanized by CO_2_ asphyxiation followed by secondary cervical dislocation. All procedures and protocols were approved by the Morehouse School of Medicine Institutional Animal Care and Use Committee.

### Temperature entrainment of endogenous period

**Fig 1:**
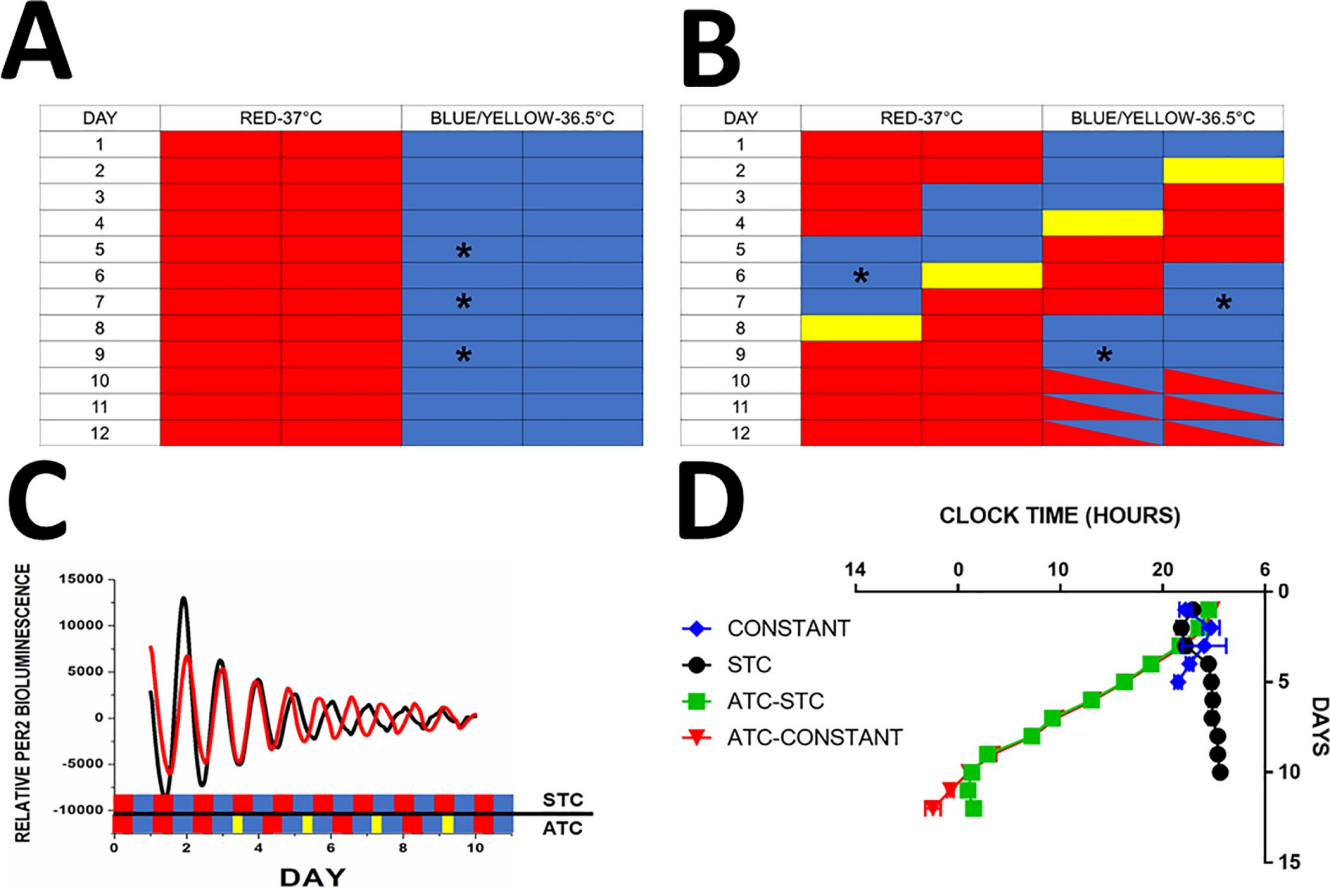
Temperature control of the splenic circadian clock. **A: Schematic illustrating static temperature cycle.** Each block represents 6 hours, red blocks are “high” temperature (37°C) while blue bars are “low” temperature (36.5°C). In experiments where immune challenge was performed, LPS was added 39 hours following the second, third, or fourth phase advance (ZT 3; Asterisks). **B: Schematic illustrating advancing temperature cycle.** Each block represents 6 hours, red blocks are “high” temperature (37°C) while blue bars are “low” temperature (36.5°C). Phase advances in the temperature cycle were accomplished by shortening the low temperature phase (yellow bars), advancing the subsequent cycle by 6 hours. Tissue was subjected to four consecutive 6-hour phase advances. In experiments where immune challenge was performed, LPS was added 39 hours following the second, third, or fourth phase advance (ZT3; Asterisks). For days 10, 11, and 12 boxes crossed with red and blue indicate where explants were either maintained in a STC or were released into constant temperature. **C: Average Bioluminescence Traces.** Traces showing relative *Per2* bioluminescence traces for spleen explants under static temperature cycle (black; STC; n = 6) and advancing temperature cycle (ATC; red; n = 6). Temperature cycle indicated beneath traces; static temperature cycle by alternating red (high temp) and blue (low temp) bars, advancing with alternating red and blue bars supplemented with shorter yellow bars indicating shortened low temp period. **D: Entrainment of Splenic Circadian Clocks by Temperature.** Peak *Per2* expression time plotted by day to demonstrate maintenance or shortening of circadian period under the following conditions: blue: kept in a constant 37°C (CONSTANT; n = 6); black: kept in a static temperature cycle (STC; n = 6) 12:12 37°C:36.5°C; green: kept in an advancing temperature cycle, followed by a static temperature cycle on day 9 after 4^th^ shift (ATC-STC; n = 6); red: kept in an advancing temperature cycle, followed by release into constant 37°C on day 9 after 4^th^ shift (ATC-CONSTANT; N = 6).

Isolated explants were maintained in temperature cycles consisting of 12 hours of a “high” (37°C) and 12 hours of “low” (36.5°C) temperature phase in temp-cycle incubators (Digitherm Incubators, Tritech Research), analogous to light:dark cycles to which animals are exposed *in vivo*. This amplitude of temperature cycle was selected because it was the smallest temperature oscillation which could entrain the tissue as well as produce type 1 phase resetting in response to shifts in the temperature cycle. Explants were then randomly assigned to either remain in the original (“static”) temperature cycle or undergo a phase advancing schedule. In order to achieve phase advances, the low portion of the temperature cycle was shortened by 6 hours every 42.5 hours for four consecutive advances (Fig 1A). Period, the length of time between adjacent daily *Per2* expression peaks at least two peaks, was calculated by monitoring the expression of *Per2*^*Luc*^ under photo-multiplier tubes (PMT Detector Assembly, Hamamatsu) inside of a temperature cycle incubator under a constant (fixed 37°C temperature; n = 6), a static temperature cycle (time of high/low temperature transitions never changed, **STC;** n = 6), an advancing temperature cycle (**ATC**) followed by placement into a STC (n = 6), and an ATC followed by release into constant temperature (n = 6; Fig 1B and 1C**)**. Type 1 phase resetting is demonstrated in Fig 1C by *mPer2* peak times on the circadian cycle following a phase advance which are intermediate between the old and new temperature cycle.

## Tissue explants

Organs were removed and micro-dissected into roughly equally sized pieces then sealed individually in 35mm dishes with 2ml of DMEM supplemented with 10mM HEPES, 2% B27, 2.5mL pen/strep, and 42μg D-(+) Glucose [8, 28, 29]. For luminometry experiments, tissue was cultured in media containing 1mM beetle luciferin (Molecular Imaging Products). Because of the phase-resetting effects of media changes no media changes were performed in while the explants were in a temperature cycle. Explants were assigned to experimental conditions at random to control for potential differences in explant size or cellular composition of individual explants.

### Lipopolysaccharide

All experiments were done as previously reported ([8, 28] purified from *Escherichia coli* serotype O111:B4 (Sigma Aldrich) was added to culture media for a final concentration of 10μl/mL. This dose was selected because previous publications have shown that this dose elicits immune responses from peritoneal macrophage culture [8], as well as from whole blood and culture PBMCs [30].

### LPS challenge protocol

We have previously reported an increase in several pro-inflammatory cytokine markers following *in vivo* ECD [8], though IL-6 was the most robust. Therefore, here we chose to use IL-6 secretion as an index of pro-inflammatory signaling. To examine the effect of shift number on the expression of *il6*, spleens were removed from the temperature cycle incubator 39 hours (ZT3) after the 2^nd^ (n = 6), 3^rd^ (n = 6), or 4^th^ (n = 6) phase advance in order to allow the tissue to complete the last shift to which they were exposed before LPS treatment. Explants were shown to complete a 6hr phase advance in just over 36 hours via analysis of bioluminescence during entrainment experiments. For all other experiments, 39 hours after the final shift at ZT 3 (or the same day/time for unshifted explants), LPS (1mM) was added to culture dishes which were then kept at 37°C for 6h for both spleen and lung explants (n = 6 for all conditions).

### qPCR

Explants were frozen in Trizol at -80°C until RNA extraction. RNA was extracted grinding explants in Trizol using a sterile pestle, then adding chloroform and spinning at 12,000 RPM for 15 mins. Supernatant is then added to isopropanol and centrifuged again to obtain RNA pellet. RNA was quantified by Specrophotometry (Nanodrop, Thermo Scientific) and standardized to 1000 ng/μl. Reverse transcription was achieved using a High Capacity cDNA Reverse Transcription Kit (Applied BioSystems) using the manufacturer recommended temperature settings for amplification. For qPCR, samples were amplified in triplicate using SsoAdvanced Universal SYBR Green Supermix (Bio Rad) and run through 39 cycles of 3 mins 10 secs at 98°C, 30 seconds of 60°C in a CGX96 Real-Time System (BioRad). *Il-6* (primer, F: TAGTCCTTCCTACCCCAATTTCC, R: TGGTCCTTAGCCACTCCTTC) was amplified and reported as relative expression of transcript (**RE**) normalized to *rpl5* (F: CTCGGATAGCAGCATGAGC, R: GGAGGAAGATGAAGATGCGTA) and to unshifted, unstimulated controls by ΔΔCt with the exception of BMAL1 KO, which was normalized to the STC. *Rpl5* was used because in pilot experiments its expression was not induced by LPS (Unstimuled = 23.16±0.21, 1hr = 27.13±0.36, 3hr = 26.07±0.28, 5hr = 26.09±0.22, 7hr = 26.13±0.36, 9hr = 26.07±0.23, 11hr = 26.28±0.29; F_6,35_ = 1.844; DF = 6; p = 0.12).

### Cytokine release

39 hours after the fourth phase advance at ZT3, tissue explants were transferred to fresh 35mm dishes with new media supplemented with LPS (n = 6) or PBS (n = 6) for 24 hours at which time an aliquot of the media was collected and frozen for analysis. Secretion of IL6 was assayed using Mouse IL-6 ELISA Set (Lower detection limit: 3.8pg/mL, BD Biosciences) in triplicate and were measured using a Spectra Max M5 plate reader (Molecular Devices).

### Statistics

For all experiments, n = 6 was used. Pilot experiments determined that this was a sufficient sample size to yield statistical power. Student’s t-test or two-way ANOVA were used as indicated in the figure legends. Posthoc analysis was done using Sidak’s multiple comparisons.

## Results

### Temperature entrainment of explanted spleens

Under constant 37°C conditions, explanted spleens have a free-running period of 23.82±0.79 hours which persisted though for only 5 days (blue, Fig 1C). In contrast, when maintained in an STC, explants retained measurable daily oscillations in *mPer2* for 10 days (black), consistent with the hypothesis that temperature acts to synchronize the circadian clocks in explanted spleens. This is further supported by a lengthening of the endogenous free-running period to 24.29±1.24 under a 24-hr STC. As expected, an ATC causes the clock to run faster in explanted spleens, as measurable by the advanced peak expression time of *mPer2* bioluminescence following an advance in the temperature cycle which are intermediate between the old and new temperature cycle. Under ATC conditions, explants had an average period of 21.43±0.21 hours while shifting persists which allows explants to complete a 6hr phase advance in ~36 hours. When returned to an STC following 4 phase advances, the period lengthens to 23.10±0.33 hours compared to 21.93±0.19 hours when released into constant 37°C. Importantly, both re-entrainment (green) and free-running (red) commence from the peak time of the last shift as opposed to the peak of *per2* expression prior to shifting (Fig 1C), further suggesting resetting of the circadian clock rather than masking by temperature.

### Effect of shift number on expression of il6

**Fig 2:**
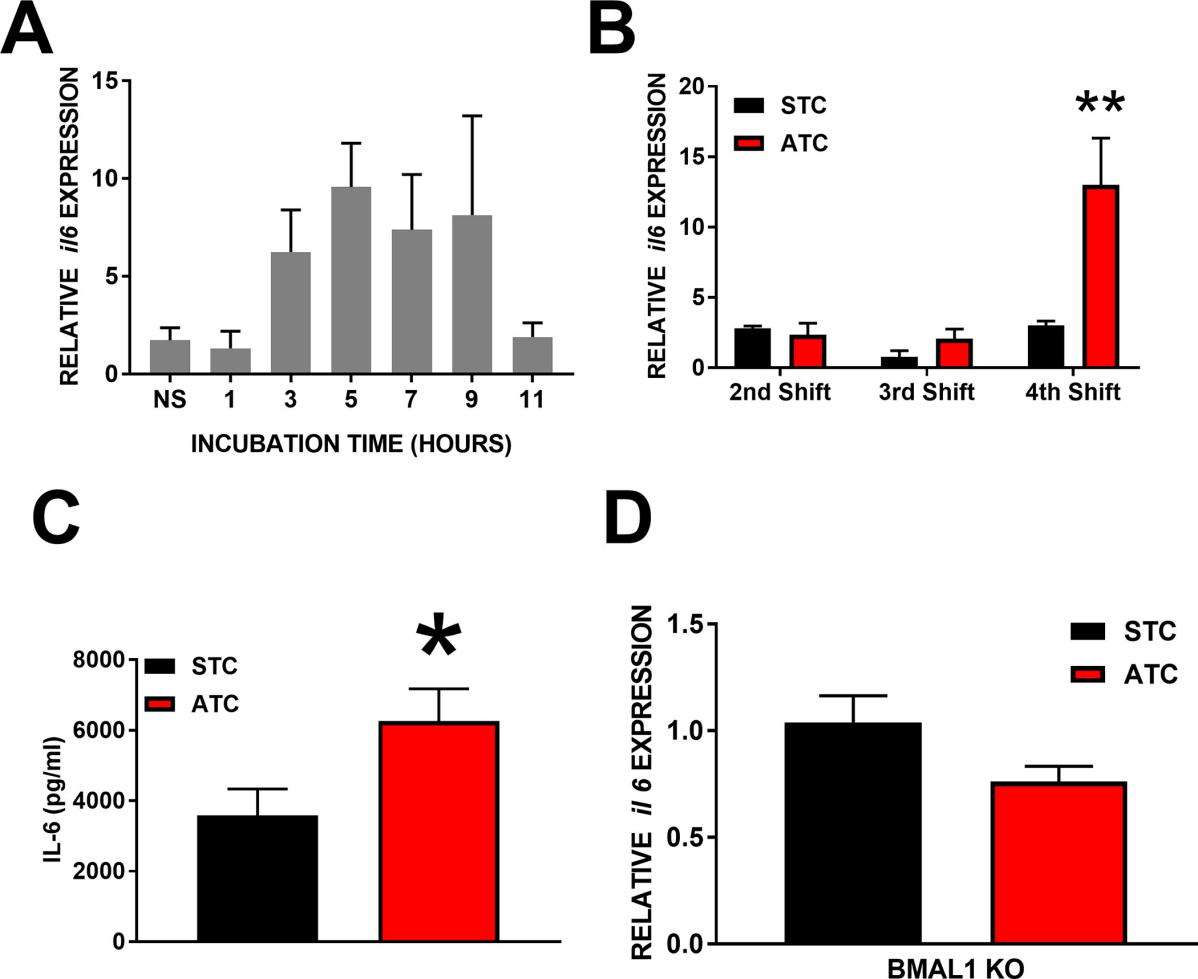
Immune consequences of circadian disruption *in vitro*. **A:** Induction of *il-6* by LPS exposure of increasing duration following four 6hr phase advances (in hours; n = 6 per condition, NS signifies not stimulated with LPS). **B:** Induction of *il-6* by LPS. Following 2, 3, or 4 six-hour phase advances (ATC; red; n = 6 per condition) or the same duration of a static temperature cycle (STC; black; n = 6 per condition), spleen explants were challenged with LPS for 6-hr. Significance determined by Two-way ANOVA. **C:** IL-6 protein secreted by spleen explants subjected to a 6-hr LPS challenge after being maintained in a static (STC; black; n = 6) or advancing (ATC; red; n = 6) temperature cycle (4 phase advances). **Significance determined by ANOVA. D:** Induction of IL-6 transcript by LPS in BMAL1KO spleen explants following either a static (STC; black; n = 6) or 4 shifts in an advancing (ATC; red; n = 6) temperature cycle (4 phase advances). Significance determined by Two-Way ANOVA (B) or Student’s T-Test (C and D). *p<0.05; **p<0.01.

Optimal LPS incubation duration was experimentally determined as being maximal after approximately 6 hours of exposure in spleen explants (Fig 2A). After an STC, LPS drove an increase in *il6* expression after the equivalent of two (2.80±0.18 RE), three (0.80±0.43 RE), or four (3.02±0.30 RE) phase advances in the ATC group, respectively. Following exposure to an ATC, LPS induced an increase in *il6* expression after two (2.36±0.81 RE), three (2.08±0.67 RE) or four (12.99±3.33 RE) phase advances. Two-way ANOVA of *il-6* induction following exposure to STC or ATC indicated that there was a main effect of both temperature cycle (p<0.05; f_2,30_ = 11.82; DF = 2) and number of shifts (p<0.05, F_2,30_ = 9.33, DF = 2), as well as a significant interaction (p<0.05, F_1,30_ = 7.47, DF = 2). Multiple comparisons revealed this was driven by increased induction of *il-6* in the ATC following the 4^th^ advance specifically (Fig 2B).

### Secretion of IL6 after 4 phase advances

The increase in *il6* was confirmed by measuring the amount of the IL6 protein in the media following the 4^th^ shift (3590.35±744.70 pg/μl vs. 6264.85±911.62 pg/μl, respectively; p<0.05; t = 2.27, DF = 10; Fig 2C).

### Effect of advancing temperature cycles with no circadian clock

Spleen explants from BMAL1KO mice exposed to an ATC did not exhibit an increase in *il6* expression (1.04±0.13 RE) compared with BMAL1KO spleen explants under STC (0.76±0.07 RE; p>0.10; t = 1.92; DF = 10; Fig 2D).

### Temperature entrainment of explanted lung

**Fig 3:**
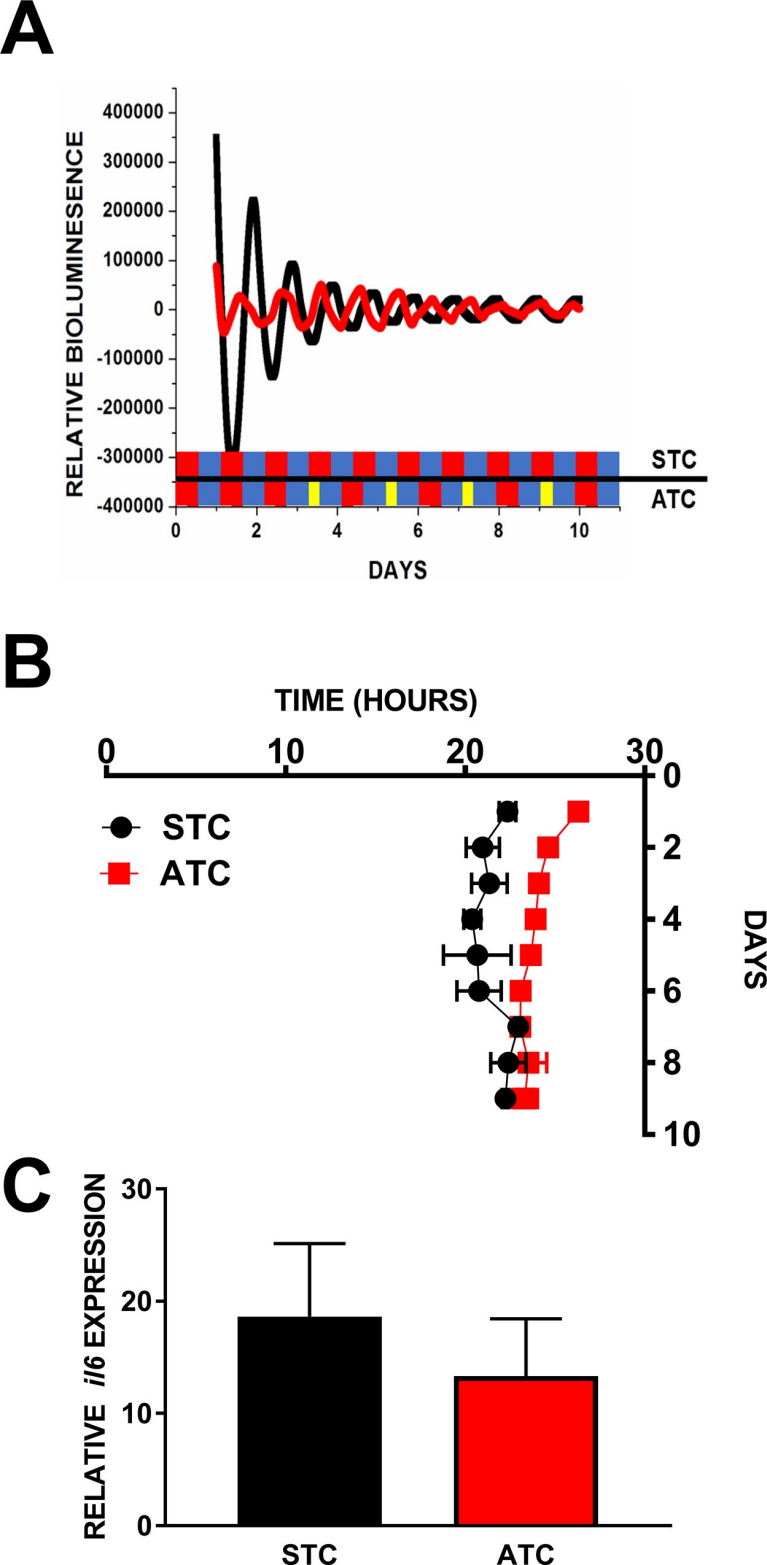
Tissue specificity of ECD in immunological function. **Lung explants are not entrained by temperature. A: Average Bioluminescence Traces.** Relative *Per2* bioluminescence traces for lung explants under static (STC; black; n = 6) and advancing (ATC; red; n = 6) temperature cycles (4 phase advances), schedule denoted in colored bars as in **Fig 1B**. **B:** Peak *Per2* expression time plotted by day to demonstrate that period in lung is not affected by a static temperature (black; STC; n = 6) or advancing temperature cycle (ATC; red; n = 6). **C:** Induction of IL-6 transcript by LPS in lung explants following either a static (black; n = 6) or advancing (red; n = 6) temperature cycle. Lack of significance was confirmed by Student’s T-Test.

In contrast to spleen explants, explanted lung tissue cultures did not appear to entrain to temperature (24.01±0.38 hours vs 24.37±0.43 hours, STC and ATC respectively; Fig 3A and 3B).

### Induction of il6 by LPS following 4 phase advances in lung

There was no difference between lung explants maintained in STC and ATC in *il-6* elicited by LPS (18.63±6.51 RE vs 13.33±5.10 RE, respectively; p>0.11; t = 0.64; DF = 10; Fig 3C).

## Discussion

### Need for a model of environmental circadian disruption

While the deleterious effects of circadian disruption have long been known, understanding how misaligning the internal clock with the external environment, however transiently, causes these effects remains obscure. Due to the inability to control for many behavioral, environmental, and genetic components of environmental circadian disruption, investigation of these causes in humans is extremely challenging. Animal models of circadian disruption allow for control of these external factors, which are very useful in understanding the difference between disruption of the relationship of the circadian clock with the environment compared with the effects of sleep loss, for example[31]. However, attempting to identify mechanisms regulating diffuse and complex processes, such as immunity, is impeded in these models by reciprocal interaction with other systems. Further, because the circadian system is organized hierarchically, with environmental input being integrated by the suprachiasmatic nucleus (SCN) of the hypothalamus and distributed to cell autonomous clocks throughout the body, identifying which levels of this hierarchy are important for mediating the effects of circadian disruption is difficult to accomplish. Much has been accomplished by using a reductionist approach in which individual clock genes are manipulated or eliminated in specific cell types to understand the role of the clock in maintaining normal physiological function. Critically however, these models do not reflect the physiological process that occurs during circadian disruption, which involves repeated, temporarily misalignment of clocks with their proximal and distal environments. Negative health outcomes in shiftwork disorders are not caused by an absent or broken circadian clock, but rather long-term, repeated exposure of a working clock to abnormal light/dark cycles. This suggests that a critical component of circadian disruption stems from a functional clock which is repeatedly establishing synchronization with the environment. In the present work, we have attempted to combine a reductionist approach with environmental circadian disruption to more closely model this repeated, temporary misalignment of the mammalian circadian system *in vivo*.

### Environmental circadian disruption in spleen explants

It has been previously shown that the molecular circadian clock in some peripheral tissues, including spleen, can be reset by temperature [24, 25]. Here, we show that simply subjecting spleen explants to an environmental temperature cycle of 0.5°C is sufficient to more than double the number of days in which a population *Per2* rhythm is detectable, compared with a static 37°C culture environment (Fig 1C). In addition, when exposed to an advancing temperature cycle, the period in *Per2*::*luc* expression is shortened such that the peak expression time occurs early on subsequent cycles (Fig 1B). Critically, when released into a non-advancing temperature cycle or a constant temperature following four consecutive shifts, the peak expression time of *Per2* 1) lengthens to readjust to environmental conditions, and 2) the phase of these oscillations begin from the phase of the last phase-advance (Fig1C). Taken together this evidence suggests real circadian phase resetting by temperature rather than masking of an unperturbed endogenous rhythm by an environmental stimulus.

### Immune dysregulation by environmental circadian disruption

It has previously been shown that the cell autonomous circadian clock in immune cells is important for their function. Immune cell trafficking and recruitment is strongly regulated by the circadian system[16, 32, 33]. Clock genes are expressed in a highly circadian manner in macrophages, which corresponds to a circadian pattern in cytokine release in response to LPS stimulation [12]. These patterns can be eliminated or shifted by deleting components of the molecular clock such as BMAL1 or REV-ERBα [13], Clock [14], and Cry1/Cry2 [15]. Here, rather than break the clock, we sought to misalign a functional circadian clock (or small suite of clocks within an organ) with phase-resetting cues in the environment, which is hypothesized to be the cause of shift work disorders [20, 34]. We have previously reported that mice subjected to 4 consecutive 6hr phase advances and then given an immune challenge in the form of LPS injection demonstrate a number of pro-inflammatory markers, notably IL6 [8]. Importantly, when peritoneal macrophages are obtained from mice subjected this shifting schedule, an exaggerated immune response is still elicited *ex vivo* [30].

Here, we demonstrate that spleens removed from unshifted mice which were subjected to ECD and LPS challenge entirely *ex vivo* show a similar increase in *il6* transcription and IL6 secretion following 4 consecutive 6hr phase advances (Fig 2B and 2C), as occurs *in vivo*. In addition, we show that this is a result of cumulative, rather than acute, circadian disruption as it is not evident after 2 or even 3 phase advances. This finding is also consistent with previous reports [8] and the etiology of shift work disorders [35, 36]. The exaggerated immune response to LPS is abolished in spleens explanted from BMAL1 KO mice (Fig 2D) which lack a circadian clock[37]. Recent work suggests that BMAL1 acts as a positive regulator of IL6 in astrocytes[38], and its loss could result in less IL6 release in response to inflammatory events. Whether this holds true in peripheral tissues such as spleen and lung is not presently known, but as IL6 production in that study was reduced rather than abolished, the lack of exaggerated *il6* induction following ECD in BMAL1 KO mice in unlikely to be caused by any regulatory role of BMAL on IL6, but rather to reflect that the repeated shifting of the clock is responsible for the exaggerated response. Thus, though the loss of timing information from the SCN rapidly results in arrhythmicity of most behavioral and physiological processes [39–41], true circadian disruption can be executed in a reductionist model if an appropriate environmental cue is used. Interestingly, these results suggest that the SCN is not necessary for the development of misalignment, or the consequence of that misalignment, between the environment and peripheral oscillators. Further, because it is known that the SCN does independently regulate innate immune cell reactivity to LPS [42], use of a reductionist model in conjunction with *in vivo* studies can tease apart the roles of local vs global contributions to circadian disruption that occurs at the organismal level. Though we acknowledge that the spleen is a heterogenous aggregation of cell types and that we have not identified specific cell-types responsible, these results provide compelling evidence that environmental circadian disruption can be successfully applied to a reduced model of immune function.

### Tissue-specific susceptibility to temperature as an entrainment signal

Having demonstrated that explanted spleens are amenable to circadian disruption using temperature as an environmental cue, we next wanted to evaluate whether this was generally true of mammalian tissue. The lung has strong *Per2*^*Luc*^ rhythms *ex-vivo* [24] and contains a large number of innate immune cells which are highly reactive to bacterial endotoxin [43, 44]. Here we were able to observe strong daily rhythms in *Per2* from explanted lung tissue but were unable to observe any change in *Per2* expression in response to an advancing temperature cycle (Fig 3A and 3B). This contrasts with previous work showing the lung capable of shifting phase in response to temperature pulses [24]. However, in that study temperature pulses of 2.5°C (36–38.5°C) were used which elicited strong phase resetting in all tissues suggesting such a large pulse acts as a very powerful entraining cue. In the present work, we found that 0.5°C temperature oscillations (37–36.5°C) were sufficient to induce phase resetting of explanted spleens such that transient peaks were observed on subsequent cycles during re-entrainment indicating perhaps a physiologically relevant environmental cue [45]. Taken together, these studies support the conclusion that while the circadian clock in lung cells can be affected by large temperature changes, it is less sensitive than the spleen to temperature as an entrainment cue. Such differential sensitivity allowed us to test whether the clock actually needs to shift in response to the environmental signal in order to produce such consequences. In contrast with spleen explants, following four 6-hr phase advances in temperature, when lung explants were challenged with bacterial endotoxin there was no difference in *il-6* induction when compared with explants maintained in a non-advancing temperature cycle (Fig 3C). The lung is composed of diverse cell types with a variety of inflammatory profiles which were not identified or sorted here but could dramatically affect the LPS response, though a useful population response was obtained from randomized explants in order to minimize the impact of the lung’s physiology on the present results. This is important because it suggests that the presence of a functional circadian clock alone is not sufficient to ensure dysregulated immune function in the presence of ECD, the clock needs to be forced to shift. At present it is not known whether temperature is an important entraining cue *in vivo*, and additional work is required to conclusively demonstrate a potential role of differential temperature sensitivity of peripheral oscillators. If true, it would provide evidence for a complex network of peripheral oscillators which have their own milieu of entrainment signals which determine the timing of their activity.

## Conclusions

In the present study we have demonstrated that at least some of the *in vivo* effects of circadian disruption on immune function can be recapitulated using a reductionist model of environmental circadian disruption. Given the importance of understanding how circadian disruption negatively impacts physiology, this model, particularly used in conjunction with *in vivo* studies, could be an important tool to identify mechanisms of circadian disruption at the cellular and organismal level. Importantly, because the organotypic culture approach in the present work did not allow for the identification of cell types or putative mechanisms involved in mediating these effects, future work should take this model even further and address these questions in a cell-type specific manner.
